# Characterizing Drug use Typologies and Their Association with Sexual Risk Behaviors: A Latent Class Analysis Among Men who have Sex with Men in Mexico

**DOI:** 10.1007/s13178-023-00861-9

**Published:** 2023-08-11

**Authors:** Angel B. Algarin, Marisol Valenzuela Lara, Mauricio Hernandez-Avila, Ricardo Baruch-Dominguez, Travis Sanchez, Steffanie A. Strathdee, Laramie R. Smith

**Affiliations:** 1 Center for Health Promotion and Disease Prevention, Edson College of Nursing and Health Innovation, Arizona State University, Phoenix, AZ USA; 2 Department of Epidemiology, Emory University, Atlanta, GA USA; 3 Economic and Social Benefits, Mexican Institute of Social Security, Mexico City, MX Mexico; 4 Western Hemisphere Region, International Planned Parenthood Federation, Mexico City, MX Mexico; 5 Division of Infectious Diseases and Global Public Health, University of California San Diego, La Jolla, CA USA; 6 9500 Gilman Drive, La Jolla, CA 92093-0507 USA

**Keywords:** Mexico, Men who have sex with men, Drug use, Sexual risk behavior

## Abstract

**Introduction:**

Drug use behaviors are closely associated with increased risk for HIV and other STIs among men who have sex with men (MSM) globally. Less is known about the drug use characteristics and their association with HIV/STI risk among MSM in Mexico, who have 13 times higher risk of acquiring HIV than the general population. We characterized distinct classes of drug use behaviors among a nationwide sample of MSM in Mexico and tested their associations with HIV risk behaviors.

**Methods:**

We used latent class analysis (LCA) to analyze injection/non-injection drug use data collected by the online *Encuesta de Sexo Entre Hombres* self-administered survey among 15,875 MSM living in Mexico between May-June 2017. MSM were recruited on general social media sites (e.g. Facebook and Twitter), popular LGBT + focused web pages (e.g. Soy Homosensual and Desastre), and dating apps (e.g. Grindr and Hornet). We used robust Poisson regression to examine associations between drug use classes and recent sexual risk behaviors while adjusting for sociodemographic characteristics.

**Results:**

Most participants were under 30 years of age (65.5%), received a Bachelor’s degree or higher (65.2%), gay-identified (82.5%), HIV negative (58.1%), and lived in the Mexico City/State of Mexico region (34.5%). We identified five distinct drug use classes: Limited Drug Use (75.4%), Marijuana Only (15.1%), Sex Event Popper + Marijuana (4.3%), Club Drug + Marijuana (4.2%), and Elevated Polydrug Use (1.0%). Compared to the Limited Drug Use class, participants in all other drug use classes were significantly more likely to engage in condomless anal intercourse (aPR = 1.14–1.39; p < 0.001), sex exchange (aPR = 1.37–4.99; p < 0.001), anonymous sex (aPR = 1.22–2.01; p < 0.001), group sex (aPR = 1.50–3.28; p < 0.001), and report an STI diagnosis in the past 12 months (aPR = 1.24–2.20; p < 0.002). Estimates were largest among the Elevated Polydrug Use class.

**Conclusions:**

This study is the first to characterize drug use behaviors and HIV/STI risk among a national sample of MSM in Mexico. Understanding how distinct combinations of drug use behaviors impact sexual risk and prevention behaviors among MSM can inform how best to target and tailor future interventions to reduce HIV/STI incidence.

**Supplementary Information:**

The online version contains supplementary material available at 10.1007/s13178-023-00861-9.

## Introduction

Globally, men who have sex with men (MSM) continue to be disproportionately impacted by HIV. A scoping review of HIV and substance use in Latin America among HIV priority populations found that people who use drugs were more likely to engage in sexual risk behaviors and experience poor HIV care cascade outcomes (Huff et al., 2022). In Mexico, the prevalence of HIV among MSM is 10.8–17.4% (Algarin et al., 2023; Bautista-Arredondo et al., 2013; Vergara-Ortega et al., 2021). Previous studies have found that sexual risk behaviors such as number of sexual partners (Bautista-Arredondo et al., 2013), sex exchange (defined as giving/receiving money/goods in exchange for sex) (Vergara-Ortega et al., 2021), and lack of condom use (Bautista-Arredondo et al., 2013; Vergara-Ortega et al., 2021) were significant factors driving the HIV epidemic among national samples of MSM in Mexico. Drug use behaviors are closely associated with increased risk for HIV and other sexually transmitted infections (STIs). Identifying modifiable upstream factors, may inform strategies to reduce the risk of HIV acquisition among MSM in Mexico and meets the recommendation of the Latin American Commission on Drugs and Democracy to focus on drug use as a health problem (Latin American Commission on Drugs and Democracy, 2009).

The majority of research on HIV and substance use in Latin America has predominately focused in Brazil and lacks quality data on HIV risk groups including MSM (Huff et al., 2022). In Mexico, a national substance use report conducted by Espolea (2015; Mexican non-governmental organization defending human rights, sexual and reproductive rights, gender equality and fighting HIV/AIDS, stigma and discrimination among young people) found that 46% of MSM were using substances besides alcohol in the past year. Yet, the 2019 Mexican National Survey on the Consumption of Drugs, Alcohol, and Tobacco found that only 3.4% of men in general engaged in illegal drug use in the past year (Comsion Nacional Contra Las Addiciones (CONADIC), 2019), highlighting the disparity of drug use among sexual minority men (Baruch-Dominguez et al., 2015). There are unique structural, legal and sociocultural factors that have upstream impacts on substance use and safe access to HIV prevention services. Equaldex ((2023); a crowdsourced knowledge base tracking laws and global view of sexual and gender minorities) has found that while the country’s equality index is relatively high in comparison to the rest of the world, laws surrounding conversion therapy vary by region and public opinion of sexual and gender minority rights are low. In a 2015 report of a sample of nationally recruited LGBT+, 25.5% of gay men and 19.2% of bisexual men reported experiencing violence due to their sexual identity, specifically in being made to feel that they need to be more masculine (Mendoza et al., 2015). Moreover, fatal violence against sexual and gender minorities has increased in recent years and may be the result of increased visibility in recent years that confronts cultural norms related to machismo and the legacy of the Catholic church (Lopez, 2020). Most drug use research among MSM in Mexico has focused on specific drugs (i.e. nitrite inhalants and methamphetamine use) (Loza et al., 2020; Pepper et al., 2020) in the Mexico-U.S. border region (Loza et al., 2020; Pepper et al., 2020; Semple et al., 2017), limiting our understanding of how poly-substance use may affect HIV risk in this population nationally. For example, in the Tijuana/San Diego border region Pepper et al. (2020) found that use of nitrite inhalants (e.g. poppers) was significantly associated with trading sex for goods, though another study by Semple et al. (2017) found no significant association between illicit drug use and condomless anal intercourse (CAI) in the region. Another study by Loza et al. (2020) in the Ciudad Juarez/El Paso border region found that methamphetamine use was associated with increased proportions of transactional sex for methamphetamine among MSM in comparison to non-MSMs.

Latent Class Analysis (LCA) is a cross-sectional latent variable mixture modeling approach used to probabilistically assign participants to subgroups based on the data they provide. This method has previously been employed to classify drug use patterns among MSM in other countries (Card et al., 2018; Goldshear et al., 2023; Lim et al., 2015; Meyers-Pantele et al., 2021; Noor et al., 2021; Scholz-Hehn et al., 2022), and was recently applied to a sample of MSM in Mexico City. The recent application of LCA methods employed by Rodríguez-Bolaños et al. (2022) found that poly-substance using MSM engaged in greater sexual HIV risk behaviors, though only focused on condom use and sero-sorting in MSM accessing sexual health clinics in Mexico City. To address this gap, we characterized distinct classes of drug use behaviors among a nationwide sample of MSM in Mexico and tested their associations with sexual risk behaviors (e.g. CAI, sex exchange, anonymous sex, group sex, and if they had an STI diagnosis in the past 12 months). Moreover, we aimed to examine the association of demographic characteristics with sexual risk behaviors to better inform future prioritization of intervention.

## Materials and Methods

We conducted a secondary analysis using data collected by the *Es Entre Hombres* study, which captured valid responses from a total of N = 15,875 MSM living in Mexico between May-June 2017. Details regarding this self-administered nationwide online survey have been previously published (Baruch-Domínguez et al., 2022). In brief, MSM were recruited using popular social networking applications, popular LGBT + focused web pages, and dating apps identified by focus groups of MSM including Facebook, Twitter, Soy Homosensual, Desastre, Grindr, Hornet. Eligibility criteria included identifying as a cisgender man, 18 + years of age, previous oral/anal sex with another man, and Mexican residence. After obtaining electronic informed consent, all variables were self-reported by participants. No compensation was provided for participation in this study. The study was approved by the Ethical Committee at the National Institute of Public Health in Mexico and institutional research review boards of Emory University and the University of California San Diego.

### Drug Use Exposures of Interest

Any use of non-injection drugs in the past 12 months of marijuana, poppers, cocaine, chlorethyl, MDMA, painkillers (OxyContin, Vicodin, Percocet), benzodiazepines (Valium, Ativan, Xanax), methamphetamine, Hallucinogens (LSD, mushrooms), Ketamine, GHB/GBL, crack, mephedrone, heroin (smoked or inhaled), other drugs, and lifetime injection drug use (IDU) were measured. To assess non-injection drug use in the past 12 months, participants were asked, *“In the past 12 months, have you used any non-injectable drug excluding alcohol, tobacco, coffee, and those prescribed to you by a doctor?”* If the participant answered ‘no’ they were classified as not using any non-injection drugs. If the participant answered ‘yes’, they were asked about their use of marijuana in the past 12 months with the item, “*In the past 12 months, have you smoked or vaped marijuana (cannabis)?,”* and about the other non-injection drugs using the item, *“In the past 12 months, which drugs have you used? (check all that apply)”*. Lifetime IDU was measured with the item, *“In your lifetime, have you ever injected drugs with the exception of those that had been prescribed to you by a doctor?”* Response options for marijuana use and injection drug use included: ‘yes’, ‘no’, ‘prefer not to answer’, and ‘don’t know’. Participants who answered ‘prefer not to answer’ or ‘don’t know’ were classified as missing on the respective variable.

### Sexual Risk Behavior Outcomes of Interest

To assess sexual risk-taking behaviors, participants were asked if they had engaged in CAI, sex exchange, anonymous sex, group sex, and if they had an STI diagnosis in the past 12 months. If a participant answered ‘yes’ to the behavior they were coded as one for that outcome, and zero if they answered ‘no’. Participants who answered ‘I don’t know’ or ‘prefer not to answer’ were classified as missing on the respective variable.

### Sociodemographic Characteristics of Interest

Demographic items included age group (18–24, 25–34, 35–44, 45+), education level (< High School, High School degree, technical/bachelor’s degree, graduate degree), sexual identity (gay, bisexual, heterosexual, questioning/don’t know), HIV serostatus (positive, negative, unknown), and geographical region (Northwest, Northeast, Central, Mexico City/State of Mexico, West Coast, South/Southeast).

### Statistical Analysis

#### Latent Class Analysis

LCA was conducted in SAS (v.9.4; Cary, North Carolina) using *proc lca* (Lanza et al., 2007). We adopted the latent class analysis procedure from the study by Card et al. (2018) to identify patterns of drug use including drugs that were used by more than 30 participants. Model fit indices of 1-, 2-, 3-, 4-, 5-, 6-, and 7-class models including Akaike information criteria (AIC), Bayesian Information Criterion (BIC), consistent AIC (CAIC), adjusted BIC, and entropy, as well as key theoretical and conceptual considerations (e.g. interpretability of the classes and class sizes), were considered to determine the best characterization of the number of classes (Dziak & Lanza, 2016; Lanza et al., 2007). Latent class descriptions were subjectively assigned based on drug use patterns of each class in comparison to the overall sample’s drug use. Participants’ most likely class membership was then assigned using maximum posterior probabilities and the prevalence of each drug use class was summarized.

#### Sexual Risk Modeling

Frequencies and percentages were used to describe the characteristics of the sample. Chi-squared tests of independence were used assess significant bivariate associations between latent drug use class, demographic factors, and sexual risk factors. A Poisson regression with robust variance models were used to estimate prevalence ratios between latent drug use class and demographic factors on CAI, sex exchange, anonymous sex, group sex, and STI diagnosis in the past 12 months. We leveraged adjusted Poisson regression models with robust error variance as opposed to logistic regression as they provide more stable estimates when the outcome is common (i.e., > 10%) (Zou, 2004). Alpha (α) was set to 0.05.

## Results

After removing participants that could not be classified due to missing drug use data (n = 633, 4.0%), we were left with a final analytic sample of n = 15,242. Those who were removed were younger (χ^2^ = 22.5, p < 0.001), high school/ technical degree educated (χ^2^ = 26.6, p < 0.001), had an unknown HIV serostatus (χ^2^ = 53.7, p < 0.001), and resided in the Northwest and Northeast regions (χ^2^ = 19.2, p = 0.002). Most participants were under 30 years of age (65.5%), received a Bachelor’s degree or higher (65.2%), gay-identified (82.0%), HIV negative (58.1%), and lived in the Mexico City/State of Mexico region (34.5%). When asked about sexual risk behaviors in the past 12 months, 59.7% reported recent CAI, 11.1% reported sex exchange, 26.1% reported anonymous sex, 16.3% reported group sex, and 10.1% reported an STI diagnosis (Table 1).

**Table 1:** Sample characteristics by drug use class among men who have sex with men in Mexico (n = 15,242) – Encuesta de Sexo Entre Hombres, 2017

	Total	Class 1: Limited Drug Use	Class 2: Only Marijuana	Class 3: Sex Event Popper + Marijuana	Class 4: Club Drug + Marijuana	Class 5: Elevated Polydrug use	χ^2^; p
	N (%)	n (%)	n (%)	n (%)	n (%)	n (%)	
	15,242 (100.0)	11,488 (75.4)	2,303 (15.1)	659 (4.3)	638 (4.2)	154 (1.0)	
**Demographics**							
Age							260.1; p < 0.001
18–24 years	5,963 (39.1)	4,447 (74.6)	1,069 (17.9)	151 (2.5)	266 (4.5)	30 (0.5)	
25–29 years	4,021 (26.4)	2,945 (73.2)	630 (15.7)	186 (4.6)	203 (5.1)	57 (1.4)	
30–39 years	3,779 (24.8)	2,858 (75.6)	483 (12.8)	235 (6.2)	150 (4.0)	53 (1.4)	
40 + years	1,479 (9.70)	1,238 (83.7)	121 (8.2)	87 (5.9)	19 (1.3)	14 (1.0)	
Education Level							52.2; p < 0.001
≤Primary school	24 (0.2)	18 (75.0)	4 (16.7)	0 (0.0)	2 (8.3)	0 (0.0)	
High school/ Technical school	5,193 (34.7)	3,951 (76.1)	796 (15.3)	186 (3.6)	220 (4.2)	40 (0.8)	
Bachelor’s Degree	7,808 (52.1)	5,826 (74.6)	1,221 (15.6)	342 (4.4)	342 (4.4)	77 (1.0)	
Post-graduate	1,958 (13.1)	1,494 (76.3)	246 (12.6)	118 (6.0)	65 (3.3)	35 (1.8)	
*Missing*	259						
Sexual Identity							60.7; p < 0.001
Gay	12,432 (82.0)	9,304 (74.8)	1,884 (15.2)	597 (4.8)	510 (4.1)	137 (1.1)	
Bisexual	2,532 (16.7)	1,959 (77.4)	385 (15.2)	60 (2.4)	113 (4.5)	15 (0.6)	
Heterosexual	99 (0.7)	88 (88.9)	7 (7.1)	0 (0.0)	2 (2.0)	2 (2.0)	
Questioning/ Don’t know	99 (0.7)	75 (75.8)	15 (15.2)	0 (0.0)	9 (9.1)	0 (0.0)	
*Missing*	80						
HIV status							360.1; p < 0.001
Negative	8,514 (58.1)	6,323 (74.3)	1,351 (15.9)	366 (4.3)	403 (4.7)	71 (0.8)	
Positive	1,621 (11.1)	1,087 (67.1)	239 (14.7)	168 (10.4)	72 (4.4)	55 (3.4)	
Unknown	4,508 (30.8)	3,624 (80.4)	633 (14.0)	89 (2.0)	140 (3.1)	22 (0.5)	
*Missing*	599						
Region							102.3; p < 0.001
Northwest	1,553 (10.2)	1218 (78.4)	221 (14.2)	38 (2.5)	65 (4.2)	11 (0.7)	
Northeast	1,571 (10.3)	1,224 (77.9)	226 (14.4)	54 (3.4)	57 (3.6)	10 (0.6)	
City/State of Mexico	5,251 (34.5)	3,785 (72.1)	826 (15.7)	300 (5.7)	263 (5.0)	77 (1.5)	
Central	2,296 (15.1)	1,764 (76.8)	357 (15.6)	86 (3.8)	70 (3.1)	19 (0.8)	
West coast	2,780 (18.2)	2,100 (75.5)	426 (15.3)	104 (3.7)	129 (4.6)	21 (0.8)	
South/South East	1,791 (11.8)	1,397 (78.0)	247 (13.8)	77 (4.3)	54 (3.0)	16 (0.9)	
**Sexual Risk Behaviors** **(past 12 months)**							
Condomless anal sex							209.2; p < 0.001
No	5,249 (40.3)	4,265 (81.3)	688 (13.1)	120 (2.3)	152 (2.9)	24 (0.5)	
Yes	7,790 (59.7)	5,536 (71.1)	1,327 (17.0)	412 (5.3)	414 (5.3)	101 (1.3)	
*Missing*	2,203						
Sex exchange							487.6; p < 0.001
No	12,316 (88.9)	9,518 (77.3)	1,863 (15.1)	421 (3.4)	440 (3.6)	74 (0.6)	
Yes	1,537 (11.1)	922 (60.0)	249 (16.2)	150 (9.8)	153 (10.0)	63 (4.1)	
*Missing*	1,389						
Anonymous sex							392.1; p < 0.001
No	9,525 (73.9)	7,442 (78.1)	1,441 (15.1)	264 (2.8)	333 (3.5)	45 (0.5)	
Yes	3,361 (26.1)	2,221 (66.1)	564 (16.8)	292 (8.7)	200 (6.0)	84 (2.5)	
*Missing*	2,356						
Group sex							644.3; p < 0.001
No	10,793 (83.7)	8,420 (78.0)	1,613 (14.9)	296 (2.7)	409 (3.8)	55 (0.5)	
Yes	2,100 (16.3)	1,247 (59.4)	399 (19.0)	257 (12.2)	124 (5.9)	73 (3.5)	
*Missing*	2,349						
Sexually transmitted infection diagnosis							254.2; p < 0.001
No	13,701 (89.9)	10,504 (76.7)	2,050 (15.0)	504 (3.7)	535 (3.9)	108 (0.8)	
Yes	1,541 (10.1)	984 (63.9)	253 (16.4)	155 (10.1)	103 (6.7)	46 (3.0)	
*Missing*	0						

### Latent Class Analysis

**Fig. 1:**
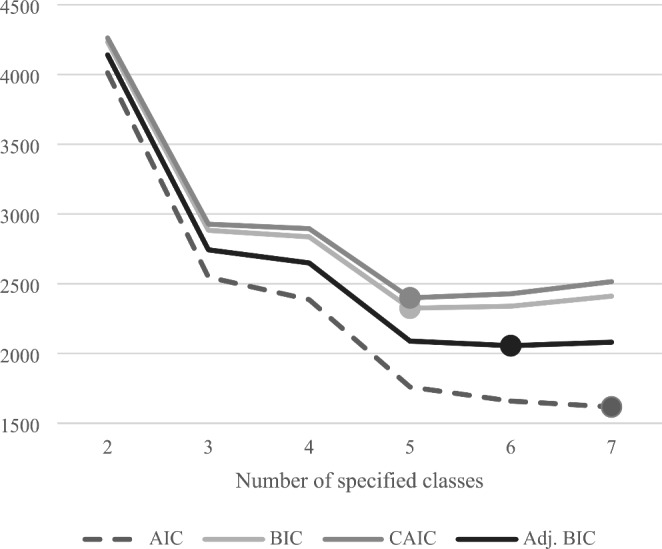
Latent Class Analysis fit statistics by number of specified classes

The most commonly used drugs among the sample were marijuana (24.0%), poppers (12.2%), and cocaine (6.9%). Mephedrone and sniffed/snorted heroin were used by less than 30 participants and were thus not included in LCA (Table 2). We compared the fit indices (AIC, BIC, CAIC, adjusted BIC) and class sizes for latent class models with 1–7 classes and chose the 5-class model for analysis. The 1–4 class models were eliminated due to poor fit, while the 6- and 7-class models were eliminated due to smaller uninterpretable class sizes (Fig. 1; Supplementary Table 1).

**Table 2:** Reported drug use among men who have sex with men in Mexico – Encuesta de Sexo Entre Hombres, 2017

Drug Options	N (%)
Marijuana	3,452 (24.0)
Poppers	1,851 (12.2)
Cocaine	1,045 (6.9)
Chlorethyl	336 (2.2)
MDMA	832 (5.5)
Painkillers	125 (0.8)
Benzodiazepines	243 (1.6)
Methamphetamine	342 (2.3)
Hallucinogens	400 (2.6)
Ketamine	104 (0.7)
GHB/GBL	70 (0.5)
Crack	99 (0.7)
Mephedrone	18 (0.4)^a^
Smoked/Snorted Heroine	0 (0.0)^a^
Other	340 (2.2)
Lifetime Injection Drug Use	172 (1.1)

a. Items with < 30 respondents

In the final 5-class solution, class 1 represented the largest group of participants (75.4%) and was characterized by low item response probabilities for all drugs and was therefore named “Limited Drug Use.” Class 2, representing 15.1% of the sample, was characterized by high item response probability for marijuana use, but low item response probability for all other drugs, and was named “Only Marijuana.” Class 3, representing 4.3% of the sample, was characterized by high item response probabilities to marijuana and popper use and was named “Sex Event Popper + Marijuana.” Class 4, representing 4.2% of the sample, was characterized by high item response probability for marijuana, cocaine, and MDMA use and was named “Club Drug + Marijuana.” Class 5, representing 1.0% of the sample was characterized by high item response probability for various drugs and was named “Elevated Polydrug Use.” Exact item response probabilities for each class can be found in Supplementary Table 2.

### Bivariate Differences by Drug Use Class

In bivariate analyses, drug use class varied by age (χ2 = 260.1; p < 0.001), education (χ2 = 52.2; p < 0.001), sexuality (χ2 = 60.7; p < 0.001), HIV-status (χ2 = 360.1; p < 0.001), and geographical region (χ2 = 102.3; p < 0.001). Additionally, drug use class varied in engagement in CAI (χ2 = 209.2; p < 0.001), sex exchange (χ2 = 487.6; p < 0.001), anonymous sex (χ2 = 392.1; p < 0.001), group sex (χ2 = 644.3; p < 0.001), and an STI diagnosis (χ2 = 254.2; p < 0.001) in the past 12 months (Table 1).

### Sexual Risk Modeling

In multivariate analyses, controlling for age, education, sexuality, HIV status, and region, we found that compared to participants in the Limited Drug Use class, participants in all other drug-using classes (i.e., Classes 2–5) were significantly more likely to engage in CAI (adjusted prevalence ratio [aPR] = 1.14–1.41; p < 0.001), sex exchange (aPR = 1.36–5.06; p < 0.001), anonymous sex (aPR = 1.22–2.29; p < 0.001), group sex (aPR = 1.50–3.46; p < 0.001), and an STI diagnosis in the past 12 months (aPR = 1.25–2.22; p < 0.002). Across drug using classes, the largest sexual risk estimates were observed among participants in the Elevated Polydrug Use class (Table 3).

**Table 3:** Adjusted prevalence ratios of recent sexual risk behaviors by drug use class and selected demographics among men who have sex with men in Mexico – Encuesta de Sexo Entre Hombres, 2017

	Condomless anal sex	Sex exchange	Anonymous sex	Group sex	Sexually transmitted infection dx
	aPR (95% C.I.) p-value	aPR (95% C.I.) p-value	aPR (95% C.I.) p-value	aPR (95% C.I.) p-value	aPR (95% C.I.) p-value
Drug Use Class					
Class 1: Limited Drug Use	Ref	Ref	Ref	Ref	Ref
Class 2: Only Marijuana	1.14 (1.10, 1.19) p < 0.001	1.36 (1.18, 1.55) p < 0.001	1.22 (1.12, 1.32) p < 0.001	1.50 (1.35, 1.67) p < 0.001	1.25 (1.10, 1.43) p < 0.001
Class 3: Sex Event Popper + Marijuana	1.37 (1.30, 1.44) p < 0.001	2.95 (2.51, 3.46) p < 0.001	1.90 (1.74, 2.09) p < 0.001	2.92 (2.61, 3.26) p < 0.001	2.02 (1.73, 2.36) p < 0.001
Class 4: Club Drug + Marijuana	1.26 (1.19, 1.33) p < 0.001	3.02 (2.59, 3.53) p < 0.001	1.55 (1.38, 1.74) p < 0.001	1.71 (1.44, 2.03) p < 0.001	1.72 (1.43, 2.07) p < 0.001
Class 5: Elevated Polydrug Use	1.41 (1.29, 1.55) p < 0.001	5.06 (4.11, 6.21) p < 0.001	2.29 (1.97, 2.66) p < 0.001	3.46 (2.91, 4.11) p < 0.001	2.22 (1.74, 2.82) p < 0.001
**Age**					
18–24 years	1.22 (1.14, 1.30) p < 0.001	0.72 (0.60, 0.86) p < 0.001	0.76 (0.68, 0.85) p < 0.001	0.78 (0.67, 0.91) p = 0.002	1.05 (0.86, 1.27) p = 0.646
25–29 years	1.21 (1.13, 1.29) p < 0.001	0.72 (0.61, 0.86) p < 0.001	0.94 (0.85. 1.05) p = 0.275	0.96 (0.83, 1.10) p = 0.519	1.28 (1.07, 1.53) p = 0.006
30–39 years	1.14 (1.07, 1.22) p < 0.001	0.75 (0.64, 0.90) p = 0.001	1.03 (0.93, 1.14) p = 0.587	0.99 (0.87, 1.14) p = 0.907	1.21 (1.01, 1.43) p = 0.036
40 + years	Ref	Ref	Ref	Ref	Ref
**Education Level**					
≤Primary school	1.22 (0.93, 1.61) p = 0.157	1.33 (0.43, 4.08) p = 0.617	0.81 (0.30, 2.22) p = 0.685	0.79 (0.20, 3.12) p = 0.738	0.59 (0.09, 4.00) p = 0.585
High school/ Technical school	1.05 (0.99, 1.11) p = 0.083	1.01 (0.86, 1.19) p = 0.888	0.95 (0.86, 1.04) p = 0.258	0.71 (0.62, 0.81) p < 0.001	0.94 (0.80, 1.10) p = 0.445
Bachelor’s Degree	1.03 (0.98, 1.08) p = 0.276	0.97 (0.84, 1.13) p = 0.705	1.01 (0.93, 1.10) p = 0.841	0.92 (0.83, 1.03) p = 0.154	1.03 (0.89, 1.18) p = 0.703
Post-graduate	Ref	Ref	Ref	Ref	Ref
**Sexual Identity**					
Gay	Ref	Ref	Ref	Ref	Ref
Bisexual	0.87 (0.83, 0.91) p < 0.001	1.25 (1.11, 1.41) p < 0.001	0.90 (0.82, 0.99) p = 0.024	0.94 (0.84, 1.06) p = 0.325	1.01 (0.88, 1.17) p = 0.844
Heterosexual	0.82 (0.65, 1.04) p = 0.099	0.88 (0.43, 1.80) p = 0.726	0.74 (0.45, 1.23) p = 0.242	0.58 (0.27, 1.27) p = 0.174	0.46 (0.16, 1.37) p = 0.165
Questioning/Don’t know	0.84 (0.68, 1.04) p = 0.110	0.74 (0.35, 1.57) p = 0.426	0.77 (0.48, 1.22) p = 0.259	0.58 (0.27, 1.21) p = 0.146	0.46 (0.15, 1.39) p = 0.946
**HIV status**					
Negative	Ref	Ref	Ref	Ref	Ref
Positive	0.99 (0.94, 1.04) p = 0.623	1.18 (1.02, 1.36) p = 0.021	1.29 (1.20, 1.40) p < 0.001	1.39 (1.26, 1.54) p < 0.001	2.63 (2.36, 2.92) p < 0.001
Unknown	0.95 (0.92, 0.98) p = 0.004	0.95 (0.85, 1.07) p = 0.426	0.79 (0.73, 0.86) p < 0.001	0.73 (0.66, 0.82) p < 0.001	0.54 (0.46, 0.63) p < 0.001
**Region**					
Northwest	1.13 (1.07, 1.18) p < 0.001	1.19 (1.00, 1.41) p = 0.053	0.54 (0.48, 0.62) p < 0.001	0.78 (0.67, 0.91) p = 0.002	1.10 (0.93, 1.30) p = 0.280
Northeast	1.07 (1.02, 1.13) p = 0.006	1.42 (1.21, 1.67) p < 0.001	0.78 (0.70, 0.87) p < 0.001	0.91 (0.79, 1.05) p = 0.203	0.94 (0.79, 1.12) p = 0.493
City/State of Mexico	Ref	Ref	Ref	Ref	Ref
Central	1.03 (0.98, 1.07) p = 0.268	1.18 (1.02, 1.37) p = 0.029	0.75 (0.68, 0.82) p < 0.001	0.88 (0.78, 0.99) p = 0.030	0.93 (0.80, 1.07) p = 0.308
West Coast	1.03 (0.99, 1.08) p = 0.132	1.11 (0.96, 1.29) p = 0.143	0.75 (0.69, 0.81) p < 0.001	0.82 (0.73, 0.92) p < 0.001	0.82 (0.71, 0.95) p = 0.009
South/South East	1.03 (0.98, 1.08) p = 0.231	1.46 (1.26, 1.70) p < 0.001	0.55 (0.49, 0.62) p < 0.001	0.81 (0.71, 0.93) p = 0.004	1.07 (0.92, 1.24) p = 0.408

Likewise, participant sociodemographic characteristics associated with sexual risk observed that those < 40 years old were more likely to engage in CAI in the past 12 months (aPR = 1.14–1.22; p < 0.001), but less likely to report sex exchange (aPR = 0.72–0.75; p ≤ 0.001) in comparison to those 40 + years of age. Additionally, those aged 18–24 were significantly less likely to have engage in anonymous (aPR = 0.76; p < 0.001) or group sex (aPR = 0.78; p < 0.001) in the past 12 months, in comparison to those aged 40 + years. Those who identified as bisexual were significantly less likely to engage in CAI (aPR = 0.87; p < 0.001) and anonymous sex (aPR = 0.90; p = 0.024) in the past 12 months, but more likely to report sex exchange for goods (aPR = 1.25; p < 0.001) in comparison to those who identified as gay. In comparison to those who identified as HIV-negative, those with unknown HIV statuses were significantly less likely to engage in CAI (aPR = 0.95; p = 0.004), anonymous sex (aPR = 0.79; p < 0.001), group sex (aPR = 0.73; p < 0.001), and were less likely to have an STI diagnosis (aPR = 0.54; p < 0.001) in the past 12 months, while those who were HIV-positive were more likely to report sex exchange (aPR = 1.18; p = 0.021), engage in anonymous (aPR = 1.29; p < 0.001) and group sex (aPR = 1.39; p < 0.001), and have an STI diagnosis (aPR = 2.63; p < 0.001) in the past 12 months (Table 3).

We also observed geographic differences, where the City/State of Mexico was significantly more likely to engage in anonymous sex (p < 0.001) in comparison to all other regions. We also found that those in the Northwest (aPR = 1.13; p < 0.001) and Northeast (aPR = 1.07; p = 0.006) regions were more likely to engage in recent CAI in comparison to those in the City/State of Mexico. Additionally, we found that those in the Northeast (aPR = 1.42; p < 0.001), Central (aPR = 1.18; p = 0.029), and South/South East (aPR = 1.46; p < 0.001) were more likely to sex exchange in comparison to the City/State of Mexico. Moreover, those in the Northwest (aPR = 0.78; p = 0.002), West coast (aPR = 0.82; p < 0.001), and South/South East (aPR = 0.81; p = 0.004) were less likely to engage in group sex (Table 3).

## Discussion

Our national sample of more than 15,000 MSM in Mexico, identified five distinct drug use classes: Limited Drug Use, Marijuana Only, Sex Event Popper + Marijuana, Club Drug + Marijuana, and Elevated Polydrug Use. While the majority of the participants were classified as Limited Drug Use, around a quarter of the sample engaged in recent drug use. Our findings highlight regional differences in drug use among MSM in Mexico, compared to national recent drug use estimates among men in the general population (3.4%) (Comsion Nacional Contra Las Addiciones (CONADIC), 2019). Given the disparities in HIV prevalence among MSM in Mexico, these results emphasize that an effective national HIV prevention strategy in Mexico must also address drug use, and related upstream determinants, associated with sexual HIV transmission and acquisition. Our results also fill gaps more broadly in Latin America by focusing in a country outside of Brazil and among an HIV priority population (Huff et al., 2022).

Our study extends previous research showing how different drug use combinations increase the likelihood of sexual risk behavior among Mexican MSM in comparison to Limited Drug Use, similar to other international studies (Achterbergh et al., 2020; Lim et al., 2015; Scholz-Hehn et al., 2022). Notably, Elevated Polydrug Use was most robustly associated with both sexual risk behaviors and living with HIV in comparison to Limited Drug Use, in line with findings from other studies (Achterbergh et al., 2020; Lim et al., 2015; Scholz-Hehn et al., 2022). This finding is particularly important for healthcare providers to consider as PrEP becomes more accessible in Mexico, as substance use among MSM in other contexts has been associated with decreased PrEP awareness (Watson et al., 2022). Efforts organized by the Mexican National Institute of Public Health should prioritize training healthcare providers in the assessment of PrEP eligibility and provide guidance on how factors such as substance use can affect PrEP engagement. Healthcare provider assessment of substance use and PrEP awareness should be prioritized to set the stage for strong PrEP rollout for those at highest risk. Moreover, status neutral HIV prevention strategies that account for elevated levels of multiple drugs known to enhance sexual experiences (i.e., poppers, cocaine, MDMA, and methamphetamine) may be most impactful in reducing HIV transmission (Loza et al., 2020; Pepper et al., 2020).

Another major finding was the significant increase in likelihood of sexual risk behaviors among MSM who only used marijuana in comparison to those with limited drug use. Though the estimated effect was smallest among this class on sexual risk behaviors, this group comprised a majority of people who used drugs in the sample. Previous studies among MSM have found mixed results on the impact of marijuana use on sexual risk behavior (D’Anna et al., 2021; Morgan et al., 2016; Passaro et al., 2019), with most recent results among Black MSM in the U.S. showing a positive association (D’Anna et al., 2021; Morgan et al., 2016). Future research is needed to disentangle the upstream effects of factors such as social stigma and discrimination on the association between marijuana use and sexual risk in minority populations. With policies changes in the near future in Mexico, following the Supreme Court rulings towards the legalization of recreational marijuana in 2021 and 2022 (McCluskey, 2021), monitoring the prevalence of marijuana use and its effects on health and behavioral outcomes among MSM is critical.

An HIV-positive serostatus was associated with reporting more overall sexual risk behaviors, though no significant differences in reporting CAI was observed. With approximately 60% of the total sample reporting recent CAI and CAI being more likely among younger MSM, it is important to consider status neutral biomedical prevention strategies focused on younger MSM, as only half of people living with HIV in Mexico are virally suppressed (UNAIDS, 2020). Interestingly, those with unknown HIV serostatus were significantly less likely to engage in sexual risk behaviors. Previous studies among MSM in Mexico suggest that those with an unknown HIV serostatus may not be engaging in sexual risk behaviors to avoid the perceived need to HIV test due potentially to fear and lack of access (Pines et al., 2016). Despite reporting fewer sexual risk behaviors, MSM who are sexually active should continue to follow the national recommendation of receiving an HIV test at least yearly, but more frequently if possible (e.g. biannually or quarterly) (DiNenno et al., 2017; Marín-Navarrete et al., 2019). Policies supporting initiatives such as HIV self-testing specifically among MSM with an unknown HIV status may help reduce barriers associated with traditional testing facilities (Valenzuela Lara et al., 2021).

Variations in sexual risk behaviors were observed across geographical regions. Differences in social and sexual norms and policies and their enforcement between states may be driving regional difference in sexual risk behaviors. In the Mexico City/State of Mexico region had higher prevalence of anonymous and group sex but lower prevalence of CAI and sex exchange in comparison to the other regions. One reason for the increased likelihood of anonymous and group sex in the Mexico City/State of Mexico region could be due to the growing underground sex scene in Mexico City among MSM (Garner, 2018). Additionally, previous research has identified the Cancun hotel zone as the “male-male sex tourism” area which could explain the increased likelihood of sex exchange in the South/South East region of Mexico compared to the Mexico City/State of Mexico region (Arroyo & Amador, 2015). In our sample, the South/South East region had the largest proportion of bisexual identified men (Baruch-Domínguez et al., 2022). Previous ethnographic research among Dominican sex workers who engage in bisexual behavior have identified touristic ecologies increasing the likelihood of sex work among this population and unique behaviors related to HIV risk (Padilla, 2008). Regional differences in sexual risk behavior should continue to be explored, particularly considering rates of sexual tourism and venue types.

We also found significant differences in sexual risk behaviors by age. Those < 40 years of age were more likely to engage in CAI, but less likely to engage in sex exchange and group sex. Findings from previous longitudinal research from the United States examining the impact of age on sexual risk taking behaviors are mixed (Dariotis et al., 2008, 2011), with some scholars positing that sexual debut and years of sexual experience being important to consider (Bozon et al., 2009; Crosby et al., 2015; Lowry et al., 2017; Nelson et al., 2016). Other scholars posit that generational sexuality policy changes (e.g. gay marriage, protections against discrimination) (Vasilenko et al., 2019), the availability of the internet for sexual identity development and education (Lozano-Verduzco & Rosales Mendoza, 2016), and the development of effective HIV treatment may also affect sexual risk behaviors as younger MSM experience a more accepting Mexican society than their older peers. Our findings suggest that refinement of safer-sexual messaging by age group and sexual risk behavior may be effective. Items surrounding age, sexual debut, and years of sexual experience should be incorporated into large national serial cross-sectional and/or longitudinal surveys to better understand the impact of these factors on sexual risk behavior in a Mexican context. Moreover, national policy should ensure resources are not exclusively concentrated in Mexico City as looking at HIV risk behaviors among MSM at a countrywide level may help better align a national response.

We also found that those who identified as bisexual were more likely to engage in sex exchange, but less likely to engage in CAI and anonymous sex. Similar to our findings, research in Latin America has found a higher proportion of those identified as bisexual engaged in sex exchange (Oldenburg et al., 2015). Moreover, touristic ecologies may more strongly promote higher proportions of non-gay identified (including bisexual) MSM to participate in sex exchange (Padilla, 2007). Policy makers should prioritize tailored HIV prevention programming to bisexual identified MSM engaging in sex exchange, particularly in touristic areas.

### Limitations & Strengths

First, all items were self-reported and subject to response bias. Second, this study did not consider contextual factors in determining sexual risk behaviors (e.g. monogamy, sero-concordance, viral suppression). Third, this study was cross-sectional meaning temporal differences could not be determined. Fourth, the results of this study may not be generalizable to MSM who do not have access to the internet. Additionally, the majority of the sample identified as gay and college educated which may underrepresent voices from non-gay identifying MSM and those who lack access to higher education. Moreover, those who did not answer drug use questions and were considered missing were significantly different from those who answered the drug use items. Despite the limitations, this study incorporated a large and geographically diverse sample of MSM in Mexico to categorize drug use and examine sexual risk behaviors in regions that have been previously understudied.

## Conclusion

Critical work needs to be done to address the relationship between drug use and sexual risk behavior as a driver of the HIV epidemic among MSM in Latin America, specifically Mexico. Significant differences in drug use classes and sexual risk behaviors should continue to be monitored as drug (e.g. legalization of marijuana use) and sexual (e.g. gay marriage, discrimination protections) policy changes are implemented and enforced in Mexico. Resource allocation and interventions tailored to drug use class-related prevalence, demographic characteristics, and sexual risks should be considered in future HIV prevention efforts (e.g. status neutral approaches, HIV self-testing, PrEP implementation).

## Electronic supplementary material

Below is the link to the electronic supplementary material.

Supplementary Material 1
