# Supplementary material for: Characterizing Drug use Typologies and Their Association with Sexual Risk Behaviors: A Latent Class Analysis Among Men who have Sex with Men in Mexico
Source: Sex Res Social Policy. 2023 Aug 11;21(4):1406–17. doi: 10.1007/s13178-023-00861-9 (PMC11634946; doi:10.1007/s13178-023-00861-9)
Supplement: Supplementary file 1 — Supplementary Material 1 [file 13178_2023_861_MOESM1_ESM.docx]

**Supplementary Table 1.** Latent Class Analysis Fit Statistics and Classes by Model

|  | **Fit Statistics** | | | | | **Class Sizes by Model** | | | | | | |
| --- | --- | --- | --- | --- | --- | --- | --- | --- | --- | --- | --- | --- |
| **#-class model** | **∆** | **AIC** | **BIC** | **CAIC** | **Adj. BIC** | **1-** | **2-** | **3-** | **4-** | **5-** | **6-** | **7-** |
| **1-** | 1.00 | 16443 | 16550 | 16564 | 16506 | 15242 (100.0%) | - | - | - | - | - | - |
| **2-** | 0.87 | 4012 | 4233 | 4262 | 4141 | 2627 | 12615 | - | - | - | - | - |
| **3-** | 0.87 | 2547 | 2883 | 2927 | 2743 | 1234 | 2540 | 11468 | - | - | - | - |
| **4-** | 0.84 | 2386 | 2837 | 2896 | 2649 | 2770 | 11465 | 459 | 548 | - | - | - |
| **5-** | 0.88 | 1760 | 2325 | 2399 | 2089 | 11488 | 2303 | 659 | 638 | 154 | - | - |
| **6-** | 0.90 | 1659 | 2339 | 2428 | 2056 | 1868 | 11273 | 210 | 490 | 1326 | 75 | - |
| **7-** | 0.91 | 1618 | 2411 | 2515 | 2081 | 2274 | 11136 | 165 | 69 | 599 | 143 | 855 |
| ∆=entropy; AIC= Akaike Information Criterion; BIC=Bayesian Information Criterion; CAIC= consistent Akaike Information Criterion; Adj. BIC= adjusted Bayesian Information Criterion | | | | | | | | | | | | |

**Supplementary Table 2.** Five-class LCA model Distribution and Probability Loadings

| **Class number** | **1** | **2** | **3** | **4** | **5** |
| --- | --- | --- | --- | --- | --- |
| **Names** | **Limited Drug Use** | **Marijuana Use** | **Sex Event Popper + Marijuana** | **Club Drug + Marijuana** | **Elevated Polydrug use** |
| **Distribution** | **N (%)** | **N (%)** | **N (%)** | **N (%)** | **N (%)** |
|  | 11,488 (75.4) | 2,303 (15.1) | 659 (4.3) | 638 (4.2) | 154 (1.0) |
| **Probability Loadings** | **%** | **%** | **%** | **%** | **%** |
| Marijuana | 0.0 | **92.8** | **75.0** | **86.0** | **88.0** |
| Poppers | 1.1 | 16.4 | **94.5** | 44.4 | **89.6** |
| Cocaine | 0.6 | 6.7 | 28.4 | **55.7** | **90.9** |
| Chlorethyl | 0.0 | 0.0 | 25.0 | 0.0 | **57.4** |
| MDMA | 0.1 | 3.1 | 17.0 | **60.7** | **88.8** |
| Painkillers | 0.0 | 0.8 | 2.3 | 7.9 | 15.4 |
| Benzodiazepines | 0.0 | 3.0 | 4.1 | 12.8 | 25.7 |
| Methamphetamine | 0.1 | 1.1 | 10.4 | 13.9 | **61.5** |
| Hallucinogens | 0.0 | 3.6 | 3.7 | 27.1 | **50.6** |
| Ketamine | 0.0 | 0.0 | 0.8 | 2.6 | 42.9 |
| GHB/GBL | 0.0 | 0.0 | 1.0 | 0.9 | 29.6 |
| Crack | 0.0 | 0.2 | 3.2 | 3.0 | 24.9 |
| Other | 0.0 | 13.6 | 1.4 | 2.8 | 3.5 |
| Injection Drug Use | 0.6 | 0.9 | 2.0 | 5.1 | 16.8 |
